# A New Mass Spectroscopy-Based Method for Assessing the Periodontal–Endodontic Interface after Intracanal Placement of Biomaterials In Vitro

**DOI:** 10.3390/jfb14040175

**Published:** 2023-03-23

**Authors:** Andreas Braun, Michael Berthold, Patricia Buttler, Joanna Glock, Johannes-Simon Wenzler

**Affiliations:** Department of Operative Dentistry, Periodontology and Preventive Dentistry, RWTH Aachen University Hospital, Pauwelsstrasse 30, 52074 Aachen, Germany

**Keywords:** periodontal–endodontic interface, endodontics, permeability, interface, leakage, high vacuum, mass spectroscopy

## Abstract

Optimizing the interface between biomaterials and dental hard tissues can prevent leakage of bacteria or inflammatory mediators into periapical tissues and thus avoid alveolar bone inflammation. In this study, an analysis system for testing the periodontal–endodontic interface using gas leakage and subsequent mass spectrometry was developed and validated using the roots of 15 single-rooted teeth in four groups: (I) roots without root canal filling, (II) roots with an inserted gutta-percha post without sealer, (III) roots with gutta-percha post and sealer, (IV) roots filled with sealer only, and (V) adhesively covered roots. Helium was used as the test gas, and its leakage rate was found by measuring the rising ion current using mass spectrometry. This system made it possible to differentiate between the leakage rates of tooth specimens with different fillings. Roots without filling showed the highest leakage values (*p* < 0.05). Specimens with a gutta-percha post without sealer showed statistically significantly higher leakage values than groups with a filling of gutta-percha and sealer or sealer alone (*p* < 0.05). This study shows that a standardized analysis system can be developed for periodontal–endodontic interfaces to prevent biomaterials and tissue degradation products from affecting the surrounding alveolar bone tissue.

## 1. Introduction

Parameters such as accuracy of fit and interactions with the existing residual tissue play decisive roles in the long-term stability and functionality of incorporated tissue replacement, and in the functioning of the hybrid structure of biological tissue and biocompatible material that has been created. The biomaterial used must be tolerated by the immune system and should initiate reparative or regenerative healing mechanisms [1,2]. If this does not happen, septic and/or aseptic disorders of healing can be expected. The unavoidable interface areas between the biological tissue and the material are a particular weak point in relation to potential healing disorders, in addition to immune defense reactions to the material used [3]. In addition, if there is a pre-existing condition that impairs blood circulation in the wound area, the prognosis for the overall therapy may be limited [4]. In this context, analysis of possible interfaces around integrated biomaterials is particularly important for patients with metabolic diseases, such as diabetes mellitus, and vascular diseases, in order to minimize disturbances in the healing process.

In endodontology and periodontology, new biocompatible materials need to be developed in such a way that they establish an adequate bond between the biomaterials used and endogenous tissue. Insufficient periodontal interfaces might otherwise cause the penetration of bacteria, necrotic tissue remnants, or inflammatory mediators that remain in the dentinal tubules in periapical tissues, leading to alveolar bone inflammation [5]. Bioactive dental glass–ceramics are suggested for dentin hypersensitivity treatment, implant coating, bone regeneration, and periodontal therapy, as they show bone-bonding ability and stimulate positive biological reactions at the material/tissue interface [6]. The possibility of a bond between bone and biomaterials is not seen as limited to bioactive materials; however, the surface topography is considered to be an essential factor for bond formation [7]. The methods used to analyze this type of interface need to respond to newly developed materials and support them with new investigation methods. Due to advances in materials development and processing, periodontal–endodontic leakage cannot be sufficiently assessed using a simple probe and now has to be assessed using more specific methods such as confocal laser scanning microscopy or microbial penetration tests [8,9]. Dye penetration tests or scanning electron microscopy, sometimes employing a replica technique, are usually used to obtain evaluable samples [10]. Analysis of passive dye diffusion is a simple technique, but it is not necessarily reliable, since influences from capillary forces, air inclusions, or dye properties such as particle size can affect the method. Other methods such as the fluid filtration method [11] and capillary flow porometry [12] use the flow of fluids through the investigated interfaces between biomaterials and dental hard substances to assess the permeability of the interface.

With the development of new materials and biocompatible endodontic sealers [13], and the continuous pursuit of better biocompatibility and durability, the requirements for adequate bonding between these materials and endogenous tissue are becoming more stringent. In order to obtain even more accurate methods for assessing the interface between biomaterials and dental hard substances, attempts have already been made to assess the permeability of an interface using leakage-induced pressure differences between the start and the end of a filled tooth root [14]. However, to the best of the authors’ knowledge, the use of a test gas, the leakage of which is quantified using mass spectrometry after passage of a test specimen, has not yet been investigated.

The objective of this study was therefore to develop an analysis system for assessing interfaces using gas leakage and subsequent mass spectrometry, testing the hypothesis that it is possible to assess the periodontal–endodontic interface with this type of system. The test was conducted in a high vacuum to allow for the assessment of even the smallest particles. High-vacuum testing was also expected to improve the quantitative evaluation, due to standardized measurement parameters. The validity of the system was then investigated by examining differently filled root canal systems, testing whether the new test system is able to detect differences between the study groups. It is assumed that if a mass spectrometry-based analysis method is positively validated, it would be possible in further studies to not only conduct measurements of leakage rates at the periodontal–endodontic interface, but also to analyze selected tissue and microbial degradation products for their potential pathological impact on the periradicular tissues.

## 2. Materials and Methods

### 2.1. Measurement Setup

**Figure 1:**
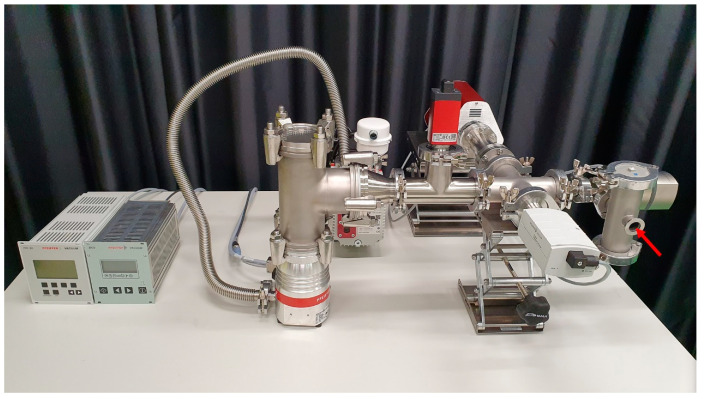
Test system for measuring the leakage of helium gas through the periodontal–endodontic interface. Red arrow: interface for the sample material.

The test setup consists of a mass spectrometer (PrismaPro QMG 250 F1ö Pfeiffer Vacuum, Wetzlar, Germany), which analyzes the test gas and displays the results via software (Figure 1). The spectrometer is designed as a quadrupole mass spectrometry system for qualitative and quantitative gas analysis in the high and ultrahigh vacuum range. It is also designed for detecting leaks and measuring trace residues. The device is mounted via a crosspiece at an angle of 90° in the gas flow between the suctioning turbomolecular high-vacuum pump (HiPace 60 P with TC 110; Pfeiffer Vacuum, Wetzlar, Germany) with a maximum volume flow of 5 × 10^−4^ hPa·l·s^−1^, and a control valve (EVR 116 gas control valve; Pfeiffer Vacuum, Wetzlar, Germany) that delivers the 99.999% high-purity test gas (Helium 5.0; Linde, Pullach, Germany). Opposite the mass spectrometer, a Pirani gauge (PKR 360; Pfeiffer Vacuum, Wetzlar) is installed to monitor the pre-vacuum pressure required for operating the spectrometer. In the event of a gas inrush, the spectrometer is shut down to prevent damage.

The backing vacuum of 2 × 10^−2^ hPa required to operate the turbomolecular pump is generated by a two-stage rotary vane vacuum pump (Duo 3M; Pfeiffer Vacuum, Wetzlar, Germany). The backing and high-vacuum pumps are connected via a metal shaft hose (ISO-KF; Pfeiffer Vacuum AG, Wetzlar). The control valve for the gas inlet into the measurement setup is controlled by a control unit (RVC 300; Pfeiffer Vacuum, Wetzlar) which, in conjunction with a Bayard–Alpert type ionization vacuum gauge, forms a control loop and ensures the constant measurement of the volume flow. The measuring principle is independent of gas type, so that incorrect measurements cannot occur when different measuring, test, or purge gases are used. The measuring tube is flanged directly into the volume flow of the measurement setup by means of a T-piece between the crosspiece of the mass spectrometer and the metering valve. A measuring chamber or sample holder can be attached to the gas inlet of the gas control valve by means of an ISO-KF flange.

### 2.2. Sample Preparation

This study included 15 freshly extracted single-rooted human teeth from different patients. Immediately after extraction, all teeth were stored in 0.9% isotonic NaCl solution with 0.001% sodium azide. This study was conducted in full accordance with established ethical principles (World Medical Association Declaration of Helsinki, version VI, 2002). All of the patients were informed that their teeth were to be used in an in vitro research project.

The roots of the teeth were separated horizontally in the apical region with a torpedo-shaped dental diamond burr (Brasseler, Lemgo, Germany) at 40,000 rpm, resulting in a residual root length of 4 mm and exposing a root canal in all cases. The root canals were checked for patency with an ISO size 10 file (K-file; VDW, Munich, Germany), and the intended root canal preparation length of 3 mm from the horizontal section level was checked using a radiograph with an ISO size 15 file inserted. Subsequently, the root canals were manually prepared up to ISO size 35 employing sodium hypochlorite 3% and ethylenediaminetetraacetic acid 17% rinsing solutions, and a check for patency was also performed again after preparation. The root specimens prepared in this way were then divided into five study groups:

- Roots without filling of the prepared root canal (positive control).
- Roots with an inserted gutta-percha post (ISO standardized gutta-percha; VDW, Munich, Germany) corresponding to the preparation size (without sealer).
- Roots with an inserted gutta-percha post corresponding to the preparation size with sealer AH Plus (Dentsply Sirona, Bensheim, Germany).
- Roots without an inserted gutta-percha post, filled with sealer only in the prepared area.
- Roots covered with a high-vacuum adhesive (IB-UHK 2020; iBEGO, Bochum, Germany) (negative control).

**Figure 2:**
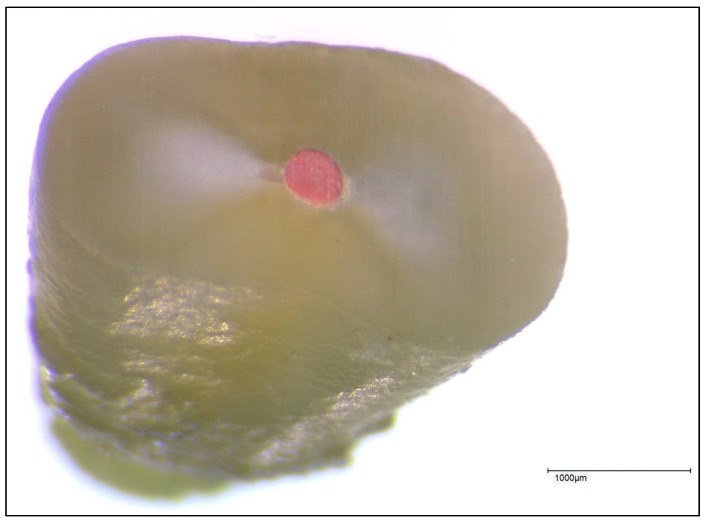
Representative image of a root specimen used for leakage measurements.

The same roots were used in the same sequence in each group. Any filling material present was completely removed from the root canal at the beginning of the experimental procedure, and the canal system was checked again for patency in each case. A representative image of a root specimen is shown in Figure 2.

For measurement of the processed specimens, sample holding devices were 3D printed in the form of an axially perforated cylinder and a trough rounded out toward the specimen. On the side facing away from the specimen, there was a tube for connecting the specimen holding device to an adapter flange for connection to the measurement setup. The specimens were fixed in the trough of the specimen holding device using a high-vacuum adhesive (IB-UHK 2020) and stored in an oven at 35 °C for 24 h to cure the adhesive. To avoid contamination of the spectrometer and vacuum system with water vapor after 24 h of water storage at 37 °C, the specimens were dried and stored in individually sealed containers with silica gel beads until measurement. For the individual measurements, the sample was placed on the adapter flange with the metering valve closed. The sample was then exposed to the helium test gas for 15 s with the metering valve open and set to a flow rate of 60 cm^3^/h. With a single measurement time of 32 ms, 469 measurements were performed within the respective measurement interval of 15 s. During the entire measurement cycle, beginning with the opening of the gas metering valve and ending with its closing, the helium spectrum after the passage of the sample was evaluated with a mass spectrometer and quantitatively displayed as ion current [A].

### 2.3. Statistical Analysis

A power analysis was performed prior to this study. The Cohen effect size was set to 0.8 [15]. For an alpha error of 0.05 and a power of 0.8, a sample size of at least 10 specimens in each group was calculated. The normal distribution of the values was assessed using the Shapiro–Wilk test. Since not all data were normally distributed, values were analyzed using a nonparametric test for dependent samples (Friedmann) and Wilcoxon pairwise comparisons. Sequentially rejective Bonferroni correction of the critical *p* value was used when multiple statistical tests were performed simultaneously on a single data set. Differences were considered statistically significant at *p* < 0.05. Box plot diagrams show the median, the first and third quartiles, and the minimum and maximum values (whiskers). Values of more than 1.5–3 times the interquartile range were specified as outliers and marked as data points. Values more than three times the interquartile range were specified as far outliers and marked as asterisks.

## 3. Results

**Figure 3:**
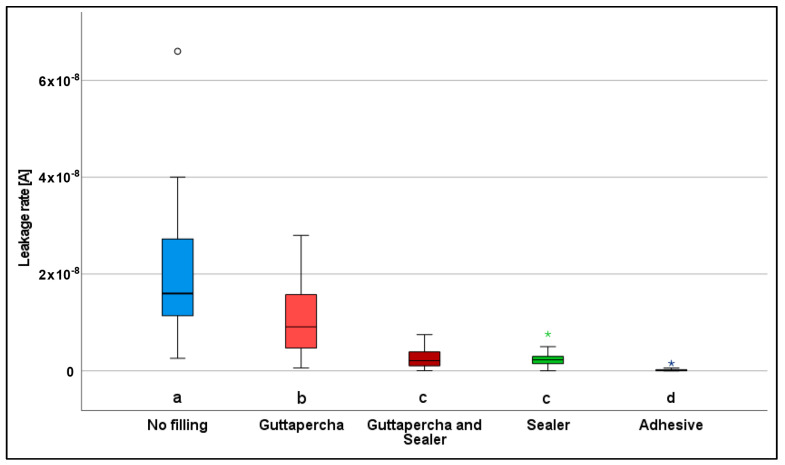
Box plot diagram for the ion current in the different study groups. Values of more than 1.5–3 times the interquartile range were specified as outliers and marked as data points “°”. Values more than three times the interquartile range were specified as far outliers and marked as asterisks “*”. Different indices (a–d) indicate groups with statistically significant differences (*p* < 0.05).

Leakage of the test gas was detected in all of the samples and measured as ion current. Statistically significantly different leakage rates were observed in the study groups investigated (*p* < 0.05) (Figure 3). Study group I (positive control) comprised the root apices of natural teeth, in which the root canal was mechanically prepared up to the physiological foramen. Since all of the preparations were tested for patency, it was ensured that an unobstructed root foramen allowed a continuous connection out of the root canal even after the area was prepared. The highest values for gas leakage among all the study groups were found in this group, with a median value of 1.4 ×10^−8^ [A] (min. 2.6 × 10^−9^, max. 6.6 × 10^−8^, interquartile range 9.4 × 10^−9^) (*p* < 0.05).

Study group II differed in that the prepared canal was obturated up to the physiological foramen with a gutta-percha post matching the preparation size without the use of a sealer. A statistically significant reduction in the leakage rate was observed in comparison with the first group, with a median value of 1.1 × 10^−8^ (min. 6.0 × 10^−10^, max. 2.8 × 10^−8^, interquartile range 1.1 × 10^−8^) (*p* < 0.05).

In study group III, the use of a sealer between the gutta-percha post and the canal wall was intended to supplement possible inaccuracies. Statistically significantly lower leakage rates were detected in this group in comparison with the first two groups, with a median value of 1.9 × 10−9 (min. 4.0 × 10^−11^, max. 7.5 × 10^−9^, interquartile range 2.6 × 10^−9^) (*p* < 0.05).

In test group IV, the filling of the root canal was performed with sealer paste alone. In comparison with groups I and II, statistically significantly lower leakage values were also observed here, with a median value of 2.0 × 10^−9^ (min. 2.0 × 10^−11^, max. 7.6 × 10^−9^, interquartile range 1.6 × 10^−9^) (*p* < 0.05). However, comparison with study group III did not show any statistically significant differences (*p* > 0.05).

In the negative controls (group V), the horizontal cutting surface of the root was covered with a high-vacuum adhesive. The statistically significantly lowest gas leakage was observed in all cases in comparison with all of the other groups, with a median value of 3.1 × 10^−11^ (min. 8.7 × 10^−12^, max. 1.6 × 10^−9^, interquartile range 2.9 × 10^−10^) (*p* < 0.05) (Table 1).

## 4. Discussion

This study investigated the development of an analysis system for testing periodontal–endodontic interfaces in a high-vacuum setting in relation to the permeation of various biomaterials into periapical tissues. It shows promising results.

Until now, leakage testing of root canal fillings or their materials could only be performed approximately using dye, bacteria, or glucose penetration tests; tests with radioactive markers; or by checking the integrity of margins using scanning electron microscopy [16]. However, these methods almost always showed very heterogeneous results, as they are too sensitive and prone to errors, among other things, due to the pH dependence of the color solution, the size of the molecule, or a lack of quantification options. Additionally, due to the semiquantitative evaluation of the results, there is often a certain lack of clarity, and therefore the corresponding significance is of extremely low clinical relevance [17,18]. In order to take these factors into account or neutralize them, this study recorded even the smallest particles purely quantitatively in a high-vacuum setting, with an option for further mass spectrometry analysis.

The dye penetration method (DPM) is the one most widely used. In this technique, the penetration depth of the dye is considered to correlate with the leakiness of the root filling [18]. It has advantages in terms of sensitivity, for example, but it is also not an accurate examination method. Another approach is the fluid filtration method (FFM), which is based on a liquid being passed through the materials to be examined, such as the sealer, resulting in cavities between the sealer and the dentin walls, and the sealer and the gutta-percha. This method was proposed many years ago as a new reference technique for leakage investigations [14,18]. Other techniques, such as capillary flow porometry (CFP), are independent of the wetting properties of the biomaterials at the interface being investigated [12]. The approach used in the present study tries to adopt the positive characteristics of the older methods, while at the same time being able to record the results and confirm their statistical significance. A similar approach, albeit on a different scale, but also using the gas permeability method (GPM), demonstrated the feasibility of this methodology to some extent [14]. However, in contrast to that study, which used nitrogen, the present study used helium as the test gas. The decision to use helium was based on the fact that it is the most commonly used gas for leak detection, and is also inert, nontoxic, and has a high diffusion capacity. As with the findings of this study, in the study previously mentioned, it became apparent how important it is to develop a method that is free of hydrophobic/hydrophilic interactions, for example, when considering biomaterials, interfaces, and leakages; in many of the methods used so far, the results are often contradictory and not comparable [17,18,19,20]. The lack of standardization seems to be just as decisive here as the variance, even within the different methods—very few results were really reproducible [21]. An interesting approach, which can perhaps be seen as a precursor and therefore not yet fully developed, investigated the possibility of leak testing using compressed air (CA), in which pore diameters of only 0.12 μm can be detected at an air pressure of 25 atm (physical atmosphere) [22]. Admittedly, the pressure values used are scarcely comparable. For compressed air, 25 atm is the equivalent of 25,331 hPa, which of course seems too high against the background of the CFP method, with up to 13,789 hPa; the fluid filtration method, with up to 1200 hPa; and the GP method, with approximately 990 hPa. However, it should be noted that the compressed air test, unlike the GP test, for example, is not an inherently closed system. The system used in the present study is also closed, with a maximum pressure difference of 900–1020 hPa between the vacuum and the environment. In comparison with the other methods, in this experimental setup it cannot be expected that a pressure constant that is too high could dissolve the materials. To confirm this again, it should be mentioned that the sample gas in the test chamber is not pressed into the samples, but gently flows around them, even with a helium supply of 60 cm^3^/h. It is diffused into the sample due to the negative pressure and is thus not a significant factor for leakage in itself. For comparison, Romieu et al. set values of 3.5 × 10^7^ mol/s or 0.5 cm^3^/h for the gas flow, but with an experimental setup that differs from ours [14].

As in the study by Romieu et al. [14], the present results also showed that varying leakage rates in the different study groups could be successfully identified. Even assuming that absorption of the test gas on the surfaces or into the depths of the samples cannot be excluded, an intraexperimental comparison of the study groups is possible, since such absorption can be assumed to be similar in all groups. Comparisons between study group I (open root canal; positive control group), study group II (obturation with gutta-percha), study group III (obturation with gutta-percha and biomaterial-based sealer), study group IV (obturation with a biomaterial-based sealer only), and study group V (adhesively sealed sample; negative control group) in most cases showed statistically significant differences. It should be added that the clear statistical significance of the positive and negative control tests confirmed that the experimental setup works and can be used without restrictions.

The different filling techniques mentioned above were also an important factor in this study, since different materials [23,24] and the layer thickness used in the techniques [25,26,27] can of course have a considerable influence on seal tightness. This was also confirmed with the method. The results show clear statistically significant differences between the control group (I) and the definitive filling groups (III and IV). However, there is still a need for further research, e.g., studies testing the seal tightness between lateral condensation, the single cone technique, and warm filling techniques or other biomaterials.

The length, root canal preparation, and irrigation of the sample must of course also be critically considered. The samples were all 4 mm in length and were repeatedly prepared (filling retreatment) as connected samples within the group. The length appears to be very short in comparison with other FF studies. Many studies, e.g., those based on the dye penetration method, use lengths of 10–15 mm [25,28]. However, this sample length was deliberately chosen for the feasibility of this study, in order to avoid influencing factors such as possible curved canals or furcations, the formation of air pockets in the root fillings, and to obtain meaningful and comparable results. Connected samples also meant that they had to be cleaned after each measurement, i.e., the filling materials had to be carefully revised. Of course, unintentional residues of materials in the samples could lead to distortions in the evaluation [29]. This can certainly also be interpreted in the results with regard to sample groups III and IV. It seems interesting that there was no statistically significant difference between the two groups and that there was a smaller interquartile range in group IV. Here, due to the missing range, one could certainly conclude that there were residues from previous filling materials from the previous run. The intention was to counteract this possibility as much as possible by using a small sample length. Visually and tactilely, an effort was made to ensure the removal of the previous biomaterial during the filling material retreatment; however, total removal of the biomaterial could not be guaranteed.

Another point that should also be considered here is the influence of the rinsing solutions used in the treatment on the adhesive strength of the biomaterials in the root canal. We used the Aachen rinsing protocol for chemomechanical preparation, which involves rinsing with a conventional 17% EDTA rinsing solution and 3% sodium hypochlorite rinsing solution. Both rinsing solutions are very well established and have been widely studied in the literature, including in connection with the filling materials used in this study, and in relation to bond strength [30]. Due to the large number of published studies on these rinsing solutions, including combinations with different types of bioactive sealants and their bonds, closer attention to this aspect seems unnecessary.

The present feasibility study provides a good insight into the analysis of interface leakage in the high-vacuum system. However, further experiments will be needed to allow more precise conclusions to be drawn. It would also be interesting to assess the extent to which different biomaterial-based (root) filling materials can prevent the leakage of sulfur compounds formed by persistent micro-organisms at the periodontal–endodontic interface, which have been described as potentially harmful [31]. There are thus exciting prospects for further research, which should definitively deal with the above-mentioned potential infiltration of toxins, using different root filling techniques and bond strengths, for example.

## 5. Conclusions

This study describes the development of a standardized test setup for assessing periodontal–endodontic interfaces in order to evaluate and optimize hybrid structures between root dentin and endodontic sealing materials. Compared to current analysis systems, it is possible not only to record leakage rates in general, but also to quantitatively measure the smallest particles, with the option of further mass spectrometric analysis. Verifying the sealing of a root canal can prevent the surrounding alveolar bone tissue from being affected by biomaterials and tissue degradation products. Determining the sealing properties of restorative biomaterials and material combinations should allow for better assessment of the prognosis for avoiding apical inflammatory processes and achieving apical healing.

## Figures and Tables

**Table 1:** Maximum ion current [A] in each study group.

	No Filling	Gutta-Percha	Gutta-Percha and Sealer	Sealer	Adhesive
Mean	2.2 × 10^−8^	1.1 × 10^−8^	2.6 × 10^−9^	2.6 × 10^−9^	2.3 × 10^−10^
Standard deviation	1.6 × 10^−8^	8.1 × 10^−9^	2.1 × 10^−9^	1.9 × 10^−9^	4.1 × 10^−10^
Median	1.4 × 10^−8^	1.1 × 10^−8^	1.9 × 10^−9^	2.0 × 10^−9^	3.1 × 10^−11^
Minimum	2.6 × 10^−9^	6.0 × 10^−10^	4.0 × 10^−11^	2.0 × 10^−11^	8.7 × 10^−12^
Maximum	6.6 × 10^−8^	2.8 × 10^−8^	7.5 × 10^−9^	7.6 × 10^−9^	1.6 × 10^−9^
Interquartile range	9.4 × 10^−9^	1.1 × 10^−8^	2.6 × 10^−9^	1.6 × 10^−9^	2.9 × 10^−10^
Number	15	15	15	15	15

## Data Availability

Data supporting the results reported here can be requested by e-mail.
